# EARLY DEVELOPMENT OF BIO-EXPERIENTIAL TECHNOLOGY TO SUPPORT PERSONS WITH DEMENTIA AND CARE PARTNERS AT HOME

**DOI:** 10.1093/geroni/igad104.2811

**Published:** 2023-12-21

**Authors:** Elizabeth Rochon, Maimouna Sy, Mirelle Phillips, Erik Anderson, Evan Plys, Christine Ritchie, Ana-Maria Vranceanu

**Affiliations:** Massachusetts General Hospital, Boston, Massachusetts, United States; Massachusetts General Hospital, Boston, Massachusetts, United States; Studio Elsewhere, Brooklyn, New York, United States; Studio Elsewhere, Brooklyn, New York, United States; Massachusetts General Hospital/ Harvard Medical School, Boston, Massachusetts, United States; Massachusetts General Hospital, Boston, Massachusetts, United States; Massachusetts General Hospital, Boston, Massachusetts, United States

## Abstract

Bio-experiential approaches offer an interactive, multisensory, immersive environmental experience using technology and physical design to promote mind-body wellness. These designs have the potential to provide an in-home solution to help persons living with dementia and their care-partners manage challenges including agitation, conflict, stress, and difficult emotions (e.g., grief). This presentation discusses a partnership between an interdisciplinary academic team and a creative design and technology studio that has pioneered bio-experiential designs. This partnership’s mission is to develop a free interactive, immersive bio-experiential platform that offers a positive and engaging experience for both persons living with dementia and their care-partner(s). Our team iteratively developed an initial prototype using multiple strategies rooted in user-centered design, including a literature review, design thinking workshop with the full team, and two focus groups with clinicians (N=10; neurologists, geriatric psychiatrists, geriatricians, neuropsychologists, social workers, occupational therapists). Early development emphasized safety to minimize risk of increased agitation or other neuropsychiatric symptoms (e.g., hallucinations, sleep impairments). Key recommendations to enhance safety and usability included simple calming visuals and sounds, personalized experiences, familiar stimuli, and multisensory engagement (e.g., music, tactile stimuli). Increasing accessibility was also discussed related to ease of use and availability of platforms (e.g., laptop, television). We are now preparing for “live” iterative usability testing of our prototype (i.e., beta testing) with a diverse group of persons living with dementia and care-partner(s) before efficacy testing. The larger goal is implementation and wide dissemination as well as adaptations for other neurodegenerative conditions. *Dr. Vranceanu and Ritchie are co-senior authors.

